# European Bison as a Refugee Species? Evidence from Isotopic Data on Early Holocene Bison and Other Large Herbivores in Northern Europe

**DOI:** 10.1371/journal.pone.0115090

**Published:** 2015-02-11

**Authors:** Hervé Bocherens, Emilia Hofman-Kamińska, Dorothée G. Drucker, Ulrich Schmölcke, Rafał Kowalczyk

**Affiliations:** 1 Fachbereich Geowissenschaften, Forschungsbereich Paläobiologie, Universität Tübingen, Hölderlinstr. 12, D-72074 Tübingen, Germany; 2 Senckenberg Center for Human Evolution and Palaeoecology (HEP), Universität Tübingen, Hölderlinstr. 12, D-72074 Tübingen, Germany; 3 Mammal Research Institute Polish Academy of Sciences, Gen. Waszkiewicza 1c, 17-230 Białowieża, Poland; 4 Centre for Baltic and Scandinavian Archaeology (ZBSA), Schloss Gottorf, D-24837 Schleswig, Germany; NYIT College of Osteopathic Medicine, UNITED STATES

## Abstract

According to the refugee species concept, increasing replacement of open steppe by forest cover after the last glacial period and human pressure had together forced European bison (*Bison bonasus*)—the largest extant terrestrial mammal of Europe—into forests as a refuge habitat. The consequent decreased fitness and population density led to the gradual extinction of the species. Understanding the pre-refugee ecology of the species may help its conservation management and ensure its long time survival. In view of this, we investigated the abundance of stable isotopes (δ^13^C and δ^15^N) in radiocarbon dated skeletal remains of European bison and other large herbivores—aurochs (*Bos primigenius*), moose (*Alces alces*), and reindeer (*Rangifer tarandus*)—from the Early Holocene of northern Europe to reconstruct their dietary habits and pattern of habitat use in conditions of low human influence. Carbon and nitrogen isotopic compositions in collagen of the ungulate species in northern central Europe during the Early Holocene showed significant differences in the habitat use and the diet of these herbivores. The values of the δ^13^C and δ^15^N isotopes reflected the use of open habitats by bison, with their diet intermediate between that of aurochs (grazer) and of moose (browser). Our results show that, despite the partial overlap in carbon and nitrogen isotopic values of some species, Early Holocene large ungulates avoided competition by selection of different habitats or different food sources within similar environments. Although Early Holocene bison and Late Pleistocene steppe bison utilized open habitats, their diets were significantly different, as reflected by their δ^15^N values. Additional isotopic analyses show that modern populations of European bison utilize much more forested habitats than Early Holocene bison, which supports the refugee status of the species.

## Introduction

The transition between the Pleistocene and the Holocene witnessed in Europe the extinction of several large mammalian herbivores, such as steppe bison *Bison priscus*, woolly mammoth *Mammuthus primigenius*, woolly rhinoceros *Coelodonta antiquitatis* and giant deer *Megaloceros giganteus*, although these species had survived there previously several similar climatic and vegetational oscillations during the Pleistocene [1], [2], [3]. At the end of the Pleistocene (c.a. 13,000 yrs BP) the area now forming Denmark, northern Germany and southern Sweden was already de-glaciated and about three thousand years later it was inhabited by survivors of the mega-herbivore community: European bison (*Bison bonasus*), aurochs (*Bos primigenius*), moose (*Alces alces*) and reindeer (*Rangifer tarandus*) [1], [4]. The earliest Holocene fossil remains of European bison, from between 12,000±600 and 10,022 ± 229 cal BP, were found in southern Scandinavia and northern Germany and this species is considered the likely niche successor (ecological replacement) of the extinct steppe bison *Bison priscus*, which was widespread in the Pleistocene [2] and exhibited high morphological similarities with modern European bison [5]. Later in the Holocene, bison and other large herbivores faced severe environmental changes, first in effect of Early Holocene forest expansion and later due to deforestation and the demographic explosion of human population related to Neolithic agriculture spread, which began around 7500 years ago [6], [7], [8]. Infrequent records dating to the following millennia show that the bison became extinct in southern Scandinavia but still occurred in Central Europe [5]. Over the last thousand years it has become a very rare species in the western part of the Central European lowlands, and by the 19^th^ century the species survived only in a few isolated Eastern European pockets. European bison eventually became extinct in the wild at the beginning of the 20^th^ century [9], but were restored to the wild from captive survivors and today occur in over 30 isolated locations in Eastern Europe [10], together with one population in Western Europe [11]. Aurochs, the second largest herbivore to survive in Europe until the Holocene, became extinct in 1627 [12]. European moose populations also experienced numerous range reductions and fragmentations in the past, but this species has returned to most of its original range in Europe during the 20^th^ century [13], [14].

The habitat of the European bison, the largest wild mammal living in Europe today, is traditionally considered to be forest. This notion comes from its survival in this type of environment during historical times [15], [16]. However, several lines of evidence suggest that European bison probably evolved in grasslands or mixed habitats [17]. These include some morphological features of the species, such as a wide muzzle, hypsodont teeth, and length of the anterior part of the jaw, which would confirm its adaptation to graze in open environments [18], as well as its dietary habits [19]. It has been proposed that bison is a refugee species and that its survival in the forest habitats may be a reaction to environmental changes and anthropogenic pressure rather than a reflection of the natural habits and food preference of the species [17], [20].

Stable isotopes next to tooth microwear analysis are powerful tools because as showed recently, morphology is not always the best indicator of the type of feeding strategies of ungulates [21], [22], [23]. It is known that carbon and nitrogen isotopic compositions of an animal body reflect the isotopic compositions of its diet and the type of foraging habitat, with a shift (isotope fractionation) whose value depends on the considered tissue [24], [25], [26], [27]. This approach has been used to document recent ecological changes in a range of species and also for the reconstruction of their diet and habitat use in the past [28], [29]. In addition, stable isotope analyses provide an unprecedented opportunity to explore the relationship between different species at present, as well as in the past, to identify interspecific resource partitioning or food and niche competition [30], [31], [32], [33], [34]. Comparison of the isotopic composition of fossil and modern material will not only provide similar information on the European bison and other large herbivores, but also may indicate the pre-refugee ecology of this large herbivore. Such insights would certainly help to improve the strategies for the conservation of this endangered species.

Investigations of herbivores' habitats in temperate and boreal ecosystems of Europe are based on the differences in δ^13^C of C3 plants growing in different environmental conditions. Since the dense understory is poorly ventilated, the decomposition of leaf litter provides depleted CO_2,_ and the intensity of light reaching the forest floor is decreased, plants growing under a shade-crown dense canopy exhibit depleted δ^13^C abundance [35], [36], in comparison to plants growing in more open habitats [37], [38], [39]. This canopy effect was observed in plants and its impact on the carbon isotopic composition in animal tissues was confirmed by investigations of modern populations of ungulates [40]. It can also be applied to the reconstruction of habitat use by animals in the past [34], [40], [41], [42], [43], [44], [45].

Both carbon and nitrogen isotopic compositions are essential for the interpretation of feeding strategy and for the tracking of competitive behavior between animals, because plant groups differ in the isotopic ratios of both chemical elements in a specific manner. As previously stated, δ^13^C values depend essentially on the density of vegetation cover. In addition, more arid conditions tend to increase the δ^13^C values of C3 plants [46]. Higher δ^15^N values in graminoids (grasses and sedges) than in shrubs and trees [30], [47], [48] allow the separation of grazing and browsing species. In boreal forest ecosystems, the δ^15^N values are higher in non- mycorrhizal plants such as graminoids, herbs, forbs, clubmosses, than in ectomycorrhizal and ericoid plants, such as trees and shrubs. Intermediate values of δ^15^N are found in mosses which, unlike most vascular plants, can rely on different sources of nitrogen, as well as in lichens [49], [50], [51], [52] (see also S1 Text). Even after standardization to a common mean annual temperature, non-mycorrhizal plants had the highest average δ^15^N [53]. The same patterns were found in open peatland, sub-arctic and arctic tundra [47], [54], [55]. Because nitrogen isotopic composition of plants may also vary due to a range of factors, such as aridity, temperature, precipitation, soil activity, maturity and acidity [48], [56], [57], investigation of herbivorous species other than bison co-occurring in the study area are needed to track differences in the diet and differentiate between browsing and grazing among herbivores.

Different studies, including those which exploit isotopic composition, have shown that the moose is today mainly a browser consuming leaves of trees and shrubs [43], [58], [59]. In turn, reindeer is known as a grazer in arctic-alpine ecosystems and also as a lichen eating species [60], [61]. Although European bison is traditionally recognized as a forest species and was re-introduced into such habitats [17], its diet is mixed, including leaves and grass, and may change seasonally [19], [62]. Extinct aurochs has been described as a grazer, especially for the Preboreal period [12], [63], [64]. Therefore, having carbon and nitrogen isotope data from coeval aurochs and moose, representing the end-members of grazing and browsing behavior respectively, will allow us to establish the level of browsing versus grazing for the bison in which we are interested.

The current study aims to reconstruct the diet and habitat utilization of the earliest Holocene European bison and accompanying species from northern Central Europe, using carbon and nitrogen isotopic compositions. We wanted to test whether European bison was originally a grazer inhabiting open habitats, as predicted by its morphological adaptations and evolutionary history, and can be considered a refugee in forest habitats.

## Materials and Methods

### Sample collection

**Fig 1:**
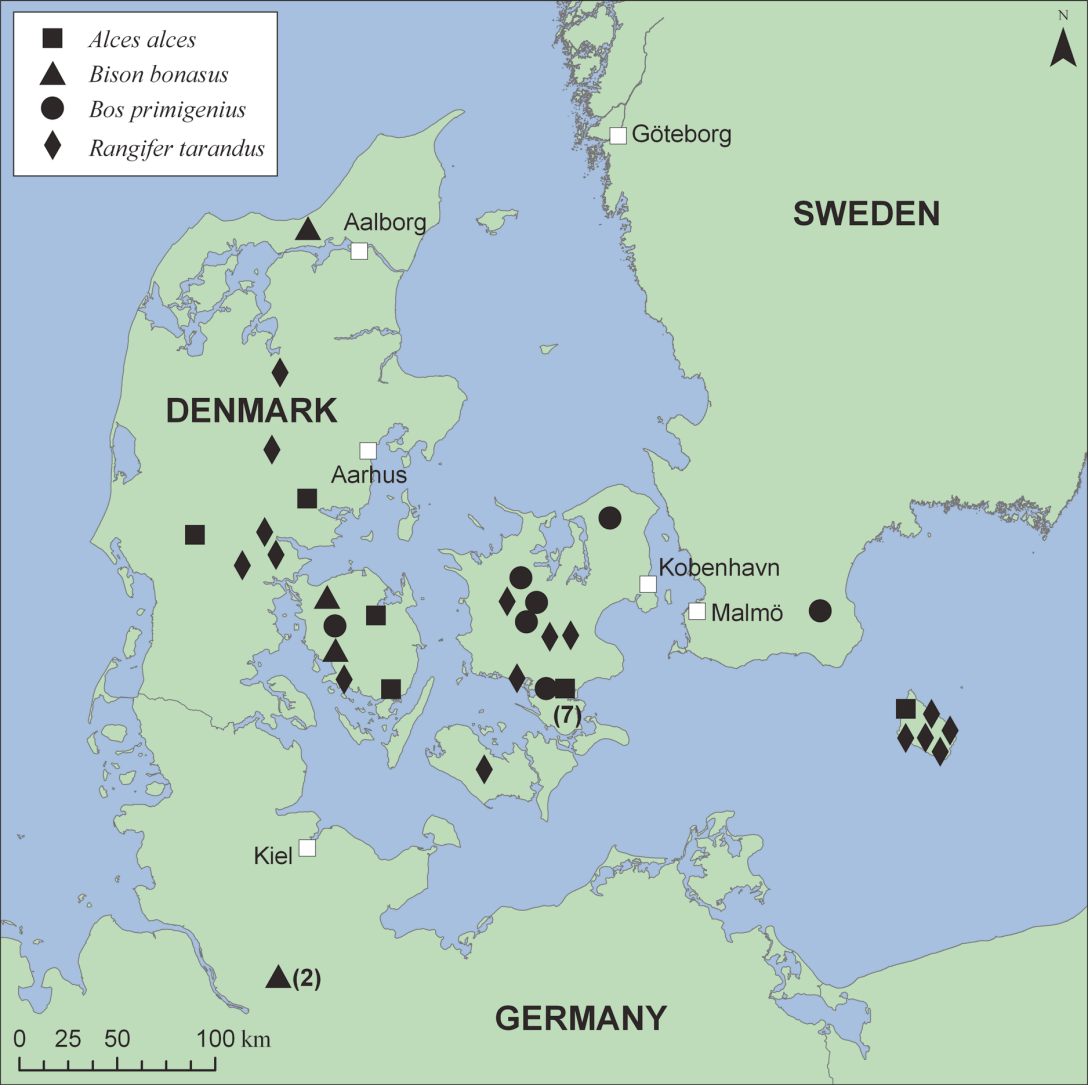
Locations of analyzed Early Holocene bone remains of *Bison bonasus*, *Bos primigenius*, *Alces alces* and *Rangifer tarandus* in northern Europe. Numbers in brackets indicate the number of individuals from the same location. The data used to generate the figure can be found in Table 1 and S1 Table.

The material used for the present study was collected from museum collections in The Natural History Museum of Denmark, University of Copenhagen (Denmark), Centre for Baltic and Scandinavian Archaeology (Germany) and Museum of Zoology, Lund University (MZLU) (Sweden), Carpathian Wildlife Research Station at Ustrzyki Dolne (Poland) and from the literature. In total five bones belonging to *Bison bonasus* were sampled for analysis of carbon and nitrogen stable isotope ratios of their bone collagen (Table 1). Four of them (two from Schleswig-Holstein in northern Germany and two from Denmark), have previously been radiocarbon dated between 10,070±50 and 8970±75 BP (11,642±229–10,022±229 cal BP) [65], [66], [67], [68]. The fifth bison bone from Denmark has been indirectly dated, from its stratigraphic context, to the same period [66] (Table 1, Fig. 1). This material represents most of the skeletal material available for the European bison in north central Europe during the early Holocene [5].

**Table 1:** Results of the isotopic analysis of the collagen extracted from bones of bison, aurochs and moose, selected from different sites in northern central Europe with information on chronology and direct datings.

**Lab-no**	**Species**	**Country**	**Site**	**Chronozone^a^**	**Age (^14^C yr BP)^b^**	**Calibrated age (cal yr BP)^c^**	**^14^C laboratory code**	**Museum collection number**	**C/N**	**%Cc**	**%Nc**	**δ^13^C (‰)**	**δ^15^N (‰)**
BIS-12	*Bison bonasus*	Germany	Stellmoor	Boreal	8970 ± 75	10,022 ± 229	OxA-3628	unknown	3.4	41.6	14.4	-20.7	4.0
BIS-13	*Bison bonasus*	Germany	Stellmoor	Younger Dryas	10,070 ± 50	11,642 ± 229	KIA-3331	unknown	3.3	41.0	14.3	-19.7	2.8
73 BP	*Bison bonasus*	Denmark	Akkerup Mose	Preboreal	9540 ± 85	10,882 ± 286	K-6005	ZMK 12/1921	3.3	39.4	13.8	-19.6	2.9
74 BP	*Bison bonasus*	Denmark	Harndrup	Younger Dryas	-	12,000 ± 600	-	ZMK 26/1944	3.3	39.4	13.9	-19.6	3.5
75 BP	*Bison bonasus*	Denmark	Jarmsted Mose	Preboreal	10,000 ± 80	11,528 ± 288	AAR-4544	ZMK 68/1944	3.4	37.5	12.7	-20.0	3.5
275 TP	*Bos primigenius*	Sweden	Frörum Mosse	Younger Dryas	10,120 ±120	11,702 ± 446	-	Lzz/3289	3.2	40.8	14.8	-22.6	6.0
77 AP	*Alces alces*	Denmark	Lunggard	Preboreal	9050 ± 125	10,160 ± 387	-	ZMK 3/1976	3.2	30. 2	10.9	-20.3	2.0
78 AP	*Alces alces*	Denmark	Tved	Preboreal	9460 ± 145	10,743 ± 442	-	ZMK 28/1990	3.3	30.0	10.7	-21.0	2.0
79 AP	*Alces alces*	Denmark	Anhoj Myr	Preboreal	9190 ± 130	10,343 ± 395	-	ZMK 177/1982	3.3	27.0	9.7	-21.4	1.5
80 AP	*Alces alces*	Denmark	Rutsker, Bornholm	Preboreal	9720 ± 135	11,134 ± 468	-	ZMK 264/1982	3.3	38.7	13.6	-21.7	3.2

^a^The following chronozones are used Younger Dryas (12,900–11,600 cal BP), Preboreal (11,600–10,640 cal. BP).

^b^The information about datings was previously published by Hedges et al. [65], Bratlund [67], Aaris-Sørensen et al. [4] and Aaris-Sørensen [66].

^c^Radiocarbon dates were calibrated using OxCal v4.2.3 with IntCal13 atmospheric curve [83], [84].

Data obtained for bison were compared with those from two other large ungulates: moose and aurochs. These bone samples were collected from Denmark and southwestern Sweden (Fig. 1) and dated from 11,702±446 cal BP to 10,160±387 cal BP ^14^C BP (Table 1). Some data on aurochs remains from the same age range (N = 5) were additionally taken from a paper by Noe-Nygaard et al. [69]. Carbon isotopic data from 16 early Holocene reindeer antlers from Denmark, published by Aaris-Sørensen et al. [4], together with seven dated moose and one dated aurochs from Denmark, published by Jessen et al. [70], have been used to provide an additional proxy of the environment (S1 Table). Reference material also included previously published isotopic data obtained from steppe bison (N = 14) and aurochs (N = 9) from Paleolithic sites located in river valleys in Belgium [71] and France [72], dated from the Late Pleistocene (S2 Table).

Early Holocene bison were also compared with material from two modern European bison populations in Poland, from the Bieszczady Mountains and Białowieża Primeval Forest, where they live in different types of forested landscape, as well as with one American bison (*Bison bison bison*) population from Prince Albert National Park in Saskatchewan, Canada (S3 Table). Original data from Bieszczady Mountains (N = 5) represent the European bison population with the highest forest utilization (70–90% of home ranges utilized) [73], [74] and low exploitation of winter supplementation. The bison population from Białowieża Primeval Forest receives fodder in the winter in the form of hay provided at feeding stations spread in the forest [9], [19], [75]. Isotopic data published already by Drucker et al. [40], for six individuals of European bison from Białowieża Primeval Forest and for nine American plains bison from Prince Albert National Park, were also used (S3 Table). The range of the Canadian plains bison includes forests (85%), meadows (10%) and water sources (5%), but bison from this population forage mainly in open meadows [76], [77].

### Sample preparation and analysis

Small samples (< 1 g) were sawn from previously identified bone remains. After cleaning with acetone and water in an ultrasound bath, the pieces were crushed to a powder of 0.7 mm grain size with a mortar and pestle and sieved. Preparation of collagen was performed following the method published by Bocherens et al. [78].

The elemental and isotopic measurements were performed at the Department of Geosciences at the University of Tübingen (Germany), using an elemental analyzer NC 2500 connected to a Thermo Quest Delta+XL mass spectrometer. The isotopic ratios are expressed using the “δ” (delta) value as follows:

$$\delta^{13}C=[\frac{{{(}^{13}C{/}^{12}C)}_{sample}}{{{(}^{13}C{/}^{12}C)}_{reference}}-1]*1000\left(‰\right)$$

and

$$\delta^{15}N=[\frac{{{(}^{15}N{/}^{14}N)}_{sample}}{{{(}^{15}N{/}^{14}N)}_{reference}}-1]*1000\left(‰\right)$$

with the international reference being V-PDB for δ^13^C values and atmospheric nitrogen (AIR) for δ^15^N values. Samples were calibrated to δ^13^C values of USGS24 (δ^13^C = -16.00‰) and to δ^15^N values of IAEA 305A (δ^15^N = 39.80‰). The reproducibility was ±0.1‰ for δ^13^C measurements and ±0.2‰ for δ^15^N measurements, based on multiple analysis of purified collagen from modern bones.

The reliability of the isotopic signatures of the collagen extracts was addressed using their chemical composition. Only extracts with %C, %N, and C/N similar to those of collagen extracted from fresh bone should be considered reliable for isotopic measurements. Several studies have shown that collagen with atomic C/N ratios lower than 2.9 or higher than 3.6 are altered or contaminated, and should be discarded [79], [80]. Extracts with 2.9 ≤ C/N ≤ 3.6 and %N < 5% may also be problematic [80] and should be excluded from further palaeobiological interpretations as well.

The δ^13^C values measured on modern American and European bison material have been corrected for the shift due to anthropogenic CO_2_ emissions using the formula δ ^13^C_atm_ = -6.429–0.0060 exp [0.0217(t-1740)] from Feng [81] and set to a δ^13^C value of atmospheric CO_2_ of-6.429‰, according to the actual date of death of the modern individuals of bison. No correction were made between Holocene and Pleistocene carbon isotopic values since the δ^13^C values of atmospheric CO_2_ have been shown recently to be comparable through the last 25,000 years until industrial development in the 19^th^ century AD [82].

All radiocarbon dates were calibrated using OxCal v4.2.3 [80] using the IntCal13 atmospheric curve [83], [84]. All dates were calibrated to BP dates with 2σ (95.4%) probability. We used non-parametric U Mann-Whitney test to verify the statistical significance of carbon and nitrogen isotopic differences (STATISTICA, StatSoft Software, Version 9.0).

## Results

**Fig 2:**
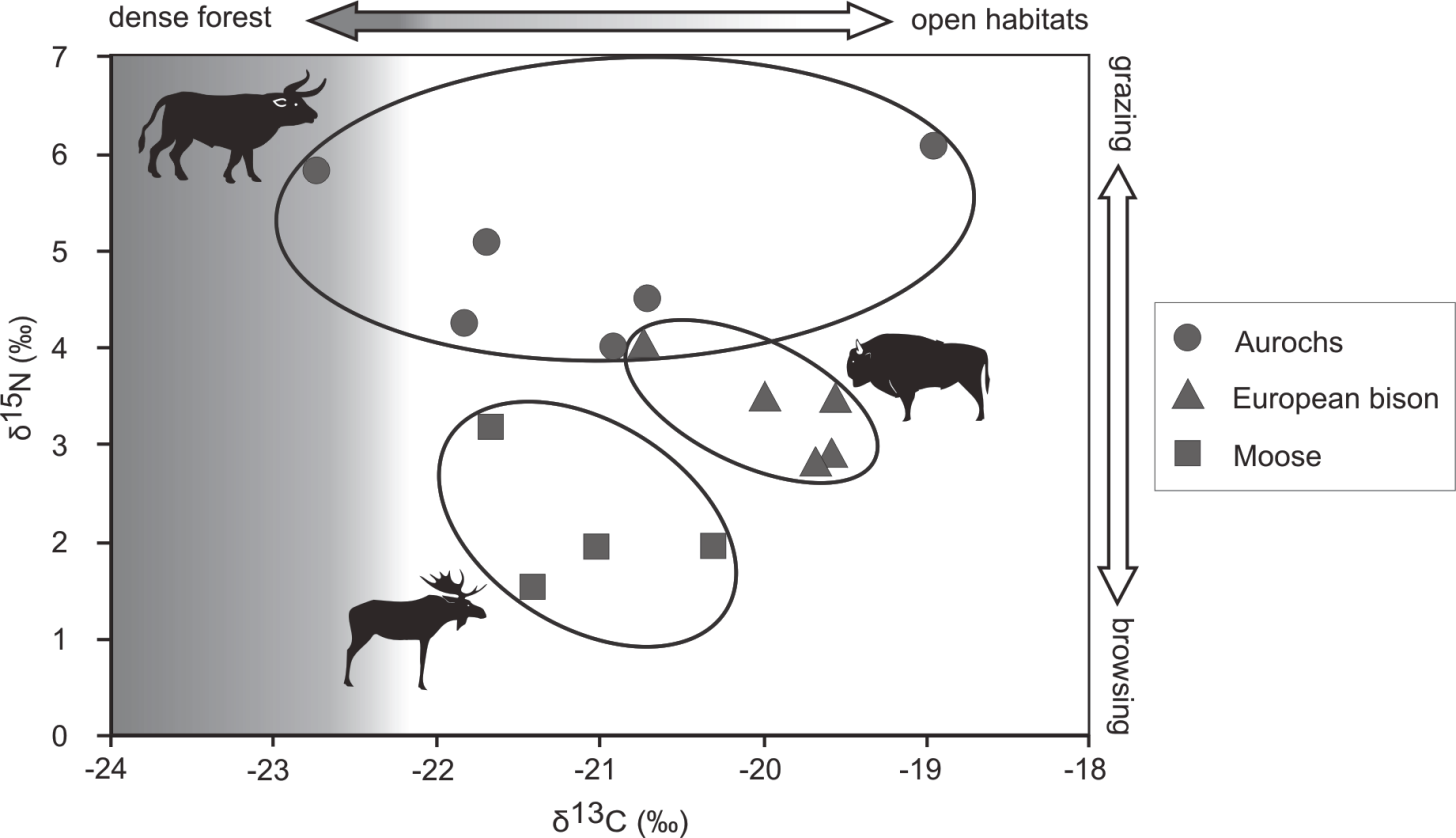
Stable δ^13^C_coll_ and δ^15^N_coll_ isotope values for moose (*Alces alces*), aurochs (*Bos primigenius*) and European bison (*Bison bonasus*) in Early Holocene in northern Europe. Shaded area denotes range of carbon isotope values characteristic for forest use. Ellipses are plotted in a way to include the extreme points of the range of measured isotopic values and to improve readability of the figure. The data used to generate the figure can be found in Table 1 and S1 Table.

**Fig 3:**
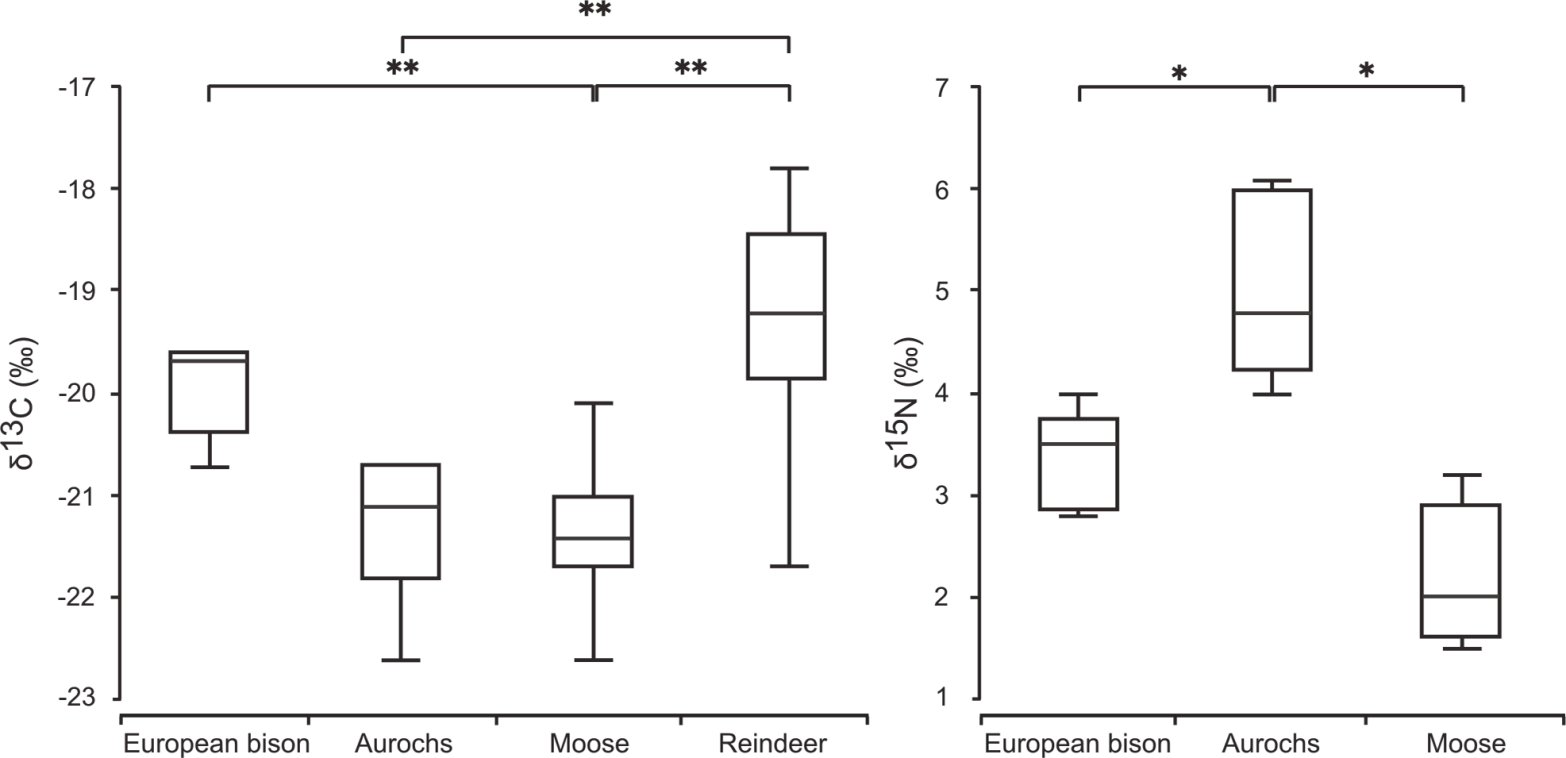
Box plots of stable δ^13^C_coll_ and δ^15^N_coll_ isotope values measured for Early Holocene moose, aurochs, European bison and reindeer. (* denote statistically significant differences for p between 0.05 and 0.01, **—for p < 0.01). Boxes show the median, upper and lower quartiles while, the whiskers show the range of the data. The data used to generate the figure can be found in Table 1 and S1 Table.

**Fig 4:**
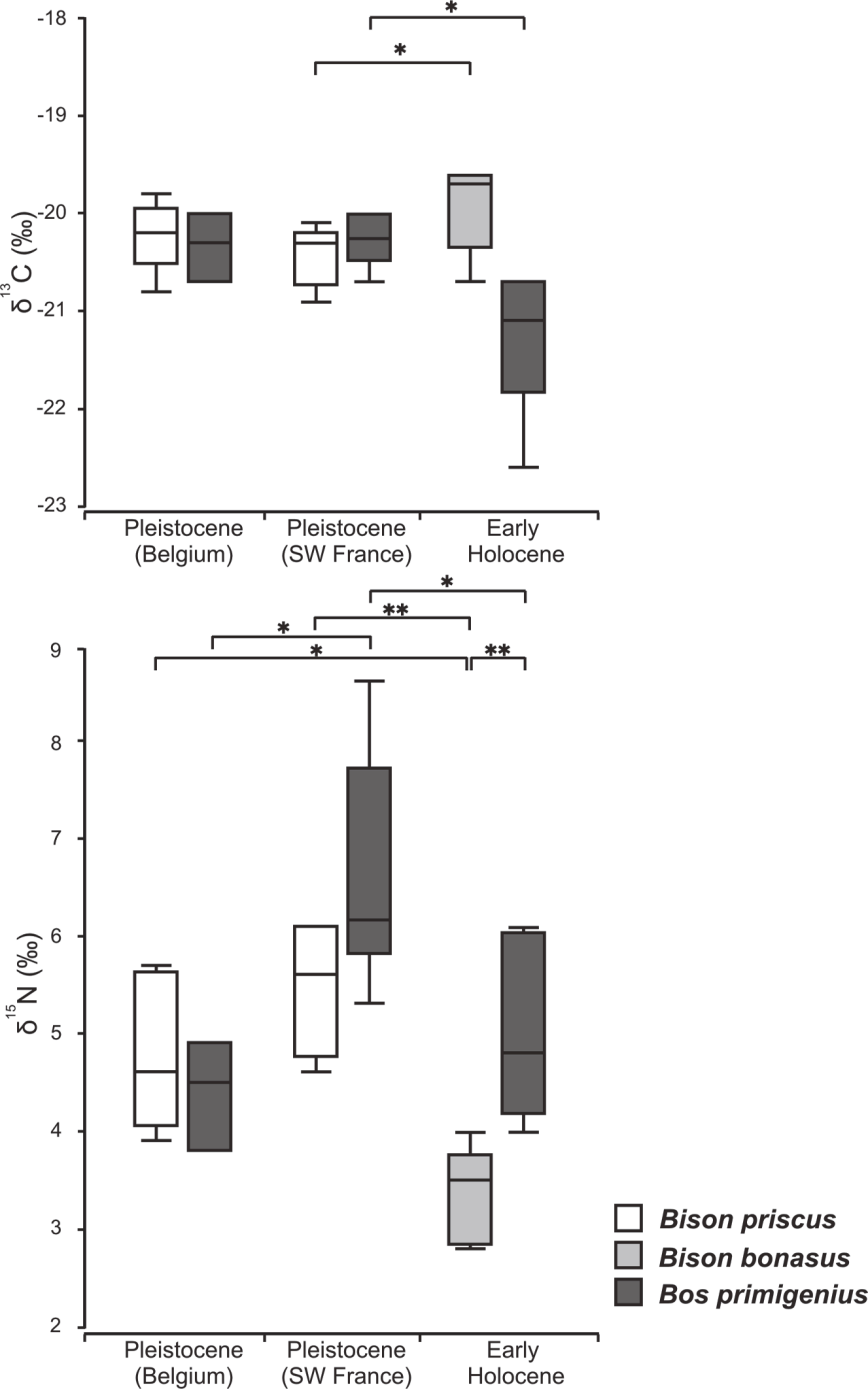
Comparison of δ^13^C_coll_ and δ^15^N_coll_ values between European bison and aurochs in Early Holocene and Pleistocene steppe bison and aurochs (Belgium and Southwestern France). (* denote statistically significant differences for p between 0.05 and 0.01, **—for p < 0.01). Boxes show the median, upper and lower quartiles, while the whiskers show the range of the data. The data used to generate the figure can be found in Table 1, S1 and S2 Tables.

The Early Holocene European bison isotopic composition ranged from -20.7 to -19.6‰ for δ^13^C and from 2.8 to 4.0‰ for δ^15^N values (Table 1, Fig. 2). For the moose, the δ^13^C values ranged from -22.6 to -20.1‰, while the δ^15^N values ranged from 1.5 to 3.2‰ (Table 1, S1 Table, Fig. 2). In the case of the aurochs, the δ^13^C values ranged widely from -22.6 to -19.0‰, while the δ^15^N values ranged from 4.0 to 6.1‰ (Table 1, S1 Table, Fig. 2). The largest data scatter, from -21.7 to -17.8‰ in δ^13^C, occurred in Holocene reindeer (S1 Table, Fig. 3). Late Pleistocene Bovinae from Belgium and France had the smallest variation in δ^13^C concentration, ranging from -19.8 to -20.9 ‰ (Fig. 4, S2 Table). Early Holocene bison and aurochs did not differ significantly in their δ^13^C values, but there were significant differences in their δ^15^N (Table 2, Fig. 3). While European bison and moose δ^15^N patterns did not differ, both species showed significant differences in concentration of δ^13^C isotopes. Early Holocene European bison and reindeer did not differ in δ^13^C values, but the latter species showed significantly higher values than aurochs and moose (Table 2, Fig. 3,). Early Holocene aurochs and moose differed in δ^15^N, being lower in bison, but not in δ^13^C (Table 2, Fig. 3).

**Table 2:** Pairwise differences for δ^13^C carbon and δ^15^N nitrogen isotope values between species in Early Holocene and Pleistocene and between samples of modern *Bison bonasus* (Poland) and *Bison bison* (Prince Albert National Park, Canada) as well as between modern and Early Holocene Bison.

****	**N:N**	**δ^13^C**	**δ ^15^N**
**EARLY HOLOCENE SPECIES COMPARISONS**			
Bos vs. Alces	6:11	0.182	**0.014**
Bison vs. Alces	5:11	**0.005**	0.064
Rangifer vs. Alces	16:11	**0.001**	-
Bos vs. Bison	6:5	0.061	**0.010**
Rangifer vs. Bos	16:6	**0.008**	-
Bison vs. Rangifer	5:16	0.098	-
**PLEISTOCENE SPECIES COMPARISONS**			
Bos vs. Bison (Belgium)	3:6	0.897	0.699
Bos vs. Bison (France)	6:8	0.366	0.061
Bos (Belgium) vs. Bos (France)	3:6	1.000	**0.028**
Bison (France) vs. Bison (Belgium)	8:6	0.366	0.107
Bison (France) vs. Bison (Early Holocene)	8:5	**0.048**	**0.004**
Bison (Belgium) vs. Bison (Early Holocene)	6:5	0.171	**0.014**
Bos (France) vs. Bos (Early Holocene)	6:6	**0.045**	**0.027**
Bos (Belgium) vs. Bos (Early Holocene)	3:6	0.137	0.305
**INTER-REGION MODERN BISON COMPARISONS**			
Bieszczady Mountains vs. Prince Albert National Park	5:9	**0.003**	-
Białowieża Forest vs. Bieszczady Mountains	6:5	0.068	-
Prince Albert National Park vs. Białowieża Forest	9:6	0.289	-
**MODERN WITH HOLOCENE BISON COMPARISONS**			
Bieszczady Mountains vs. Early Holocene Bison	5:5	**0.012**	-
Białowieża Forest vs. Early Holocene Bison	6:5	**0.008**	-
Prince Albert National Park vs. Early Holocene Bison	9:5	**0.009**	-

Insignificance at p > 0.05 is indicated in normal font for Mann-Whitney—Wilcoxon test. Bold indicates significant differences. Abbreviations: N = number of specimens.

The δ^15^N values for Holocene European bison were significantly lower than those of Late Pleistocene steppe bison but there was little, if any, difference in their δ^13^C values (Table 2, Fig. 4). In the case of aurochs, significantly lower abundance of both isotopes was recorded in Early Holocene in comparison to Pleistocene specimens from France (Table 2, Fig. 4). In contrast, no statistical differences in δ^13^C and δ^15^N isotope abundance were found between bison and aurochs from the Pleistocene, either in Belgium or France (Table 2, Fig. 4).

**Fig 5:**
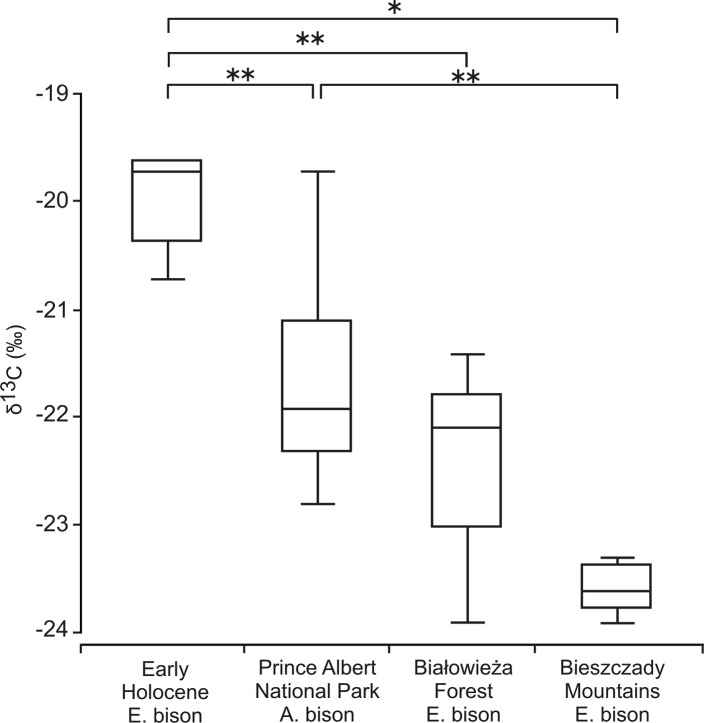
Box plots of stable δ^13^C_coll_ values for Early Holocene *Bison bonasus*, compared to modern populations of European (*Bison bonasus*) and American bison (*Bison bison bison*) living in forest habitats. (* denote statistically significant differences for p between 0.05 and 0.01, **—for p < 0.01). Boxes show the median, upper and lower quartiles, while the whiskers show the range of the data. The data used to generate the figure can be found in Table 1 and S3 Table.

Early Holocene bison showed significantly higher values of δ^13^C than the contemporary populations of European and American bison (Table 2, Fig. 5).

## Discussion

None of the carbon isotopic measurements performed on European bison, aurochs and moose indicate a dense forest habitat for these species during the Early Holocene in northern Germany and most of Denmark. However a few δ^13^C values slightly lower than -22‰ found in one aurochs from southern Sweden and two moose from Denmark reflect some early stages of forest succession. This is consistent with vegetational reconstructions that indicate relatively open landscape in Denmark and northern Germany during this period [70], [85], [86], [87]. Geological, pollen-botanical and zoological investigations have shown that northern Germany and the south of Denmark was a park tundra (steppe-tundra and forest-steppe with birch and pine), while the north-west of Denmark was covered by open tundra dominated by dwarf shrubs and willows [88]. Tundra and steppe animals such as reindeer, wild horse, aurochs and bison grazed in open conditions, which persisted throughout the first two millennia of the Holocene, especially on fine grained soils, where seasonal dryness prevented the establishment of deciduous trees [89]. However, the open forest vegetation had become attractive to forest animals including giant deer, which has been recorded from Denmark at that time [66], [88], and moose, as reflected in two of the most negative carbon isotope values (-22.6 and -22.2‰) that were measured for this species from the Preboreal, i.e. the Early Holocene period. Indeed, the beginning of the Holocene sees a global warming trend after the cold spell of the Younger Dryas and an afforestation of the former tundra landscape in the northern part of central Europe [90].

The pattern of isotopic variation among the ungulate species in northern central Europe during the Early Holocene indicates that were significant differences in habitat use and diet between the bison and the other ungulates. In contrast with the Pleistocene, when aurochs and bison strongly overlapped in their patterns of habitat use and diet, these bovines diversified ecologically as a result of the successional stages of habitats and vegetation during the early Holocene climate warming, which created a mosaic habitat with diversified vegetation. This probably allowed them to avoid competition by a separation of their food or habitat niches. Holocene bison and aurochs occupied quite similar habitats, with a wider range of habitat types for aurochs as suggested by carbon isotopes, but they differed in their respective diets. Aurochs had a more diverse diet but composed mostly of grass, while the diet of bison was more mixed. Bison and moose utilized different habitats as indicated by their δ^13^C values, but their diet as reflected in δ^15^N was quite similar, although more woody in moose. Bison may therefore be characterized as an intermediate feeder during the Early Holocene, as also observed in forest habitats now [19]. According to nitrogen isotopes, Holocene moose might be characterised as a typical browser, as also shown by microwear analyses performed on Pleistocene specimens [91]. Isotopic results are consistent with investigations of contemporary populations of these two species, as well as with δ^13^C depletion in the shrub and woody diet preferred by moose [47], [48], [58] when compared to the mixed diet of bison [62], [92]. Interestingly, when aurochs, moose and bison did not differ in the isotope concentration of one element, carbon or nitrogen, they differed in the second one. Our results show that, despite the partial overlap in carbon and nitrogen isotopic values in Early Holocene ungulates, they avoided competition by selecting different habitats or different food sources within similar habitats, a phenomenon that has also been observed among other Pleistocene ungulates [33], [34], as well as in modern populations of herbivores studied with stable isotopes [32], [93].

As expected, reindeer exhibited the least negative δ^13^C values, as reported for this species during the late Pleistocene and recent times, due to its foraging on lichen [45], [94], [95], a plant with δ^13^C values higher than those of vascular plants [96], [97], [98]. The best explanation for the scatter in Holocene reindeer being the largest among the studied herbivores is variability and seasonality in the diet of this species. At present, reindeer summer diet is composed of shrubs and includes a wide range of vascular plants and a lower share of lichen, while in winter, their diet is dominated by lichens, evergreen shrubs and mosses [94], [95], [99], [100]. The present study also confirms previously detected niche partitioning between *A*. *alces* and *R*. *tarandus* [30], [43]. Although Early Holocene bison exhibit on average higher δ^13^C values than those of most other ungulates from the same period, the fact that the δ^13^C values of bison are not significantly different from the δ^13^C values of reindeer may indicate that these bison did consume some lichens, unlike moose and aurochs. Similar food habits are observed in modern American bison in boreal forests [101], [102].

A comparison of European bison with aurochs and steppe bison can help us to better define the pre-refugee ecology of European bison. During the Pleistocene, aurochs and steppe bison occurred together at many sites, but their skeletal similarity makes them difficult to distinguish [103], [104], [105], so that remains of the two species are very often grouped together as Bos/Bison. In two cases, it was nevertheless possible to obtain isotopic values for steppe bison and aurochs in the same area at a same time, once in Southwestern France [72] and once in Belgium [71], both from times around 30,000 to 40,000 years ago. The δ^13^C values are directly comparable with those of Early Holocene bovines since recent investigations of changes in isotopic composition of atmospheric CO_2_ revealed that they are very similar between the Holocene and Pleistocene, in contrast to previous evaluations [82]. In contrast with the observed difference in δ^15^N values between bison and aurochs in the Holocene, there were no differences in their δ^15^N values when steppe bison and aurochs occurred together in the Late Pleistocene, reflecting a grazing diet for both large bovine species [71], [72]. Collagen δ^13^C values for both species were in the ranges of values characteristic for grazing on graminoids and forbs, when measured for these plants in arctic tundra [47], [48]. However, aurochs preference for a grazing diet differ from the results obtained by microwear analysis for Pleistocene specimens (45,000–34,000 BP, North Sea, Brown Bank), where this species has been recognized as a browse-dominated mixed feeder or mixed feeder, while the same study partly confirms the domination of grasses in the steppe bison diet and characterizes it as a grass-dominated mixed feeder [91]. A relatively homogeneous ecosystem, with no dense canopy, was present in the periglacial area [34] and the narrow choice of different habitats probably forced *Bison* and *Bos* to share the same dietary niche. Distinctive environmental conditions between glacial (Pleistocene) and interglacial (Holocene) periods probably reflect changing competitive relationships between co-occurring Bovinae species over the millennia.

Differences in δ^15^N values between European bison and Pleistocene steppe bison from Belgian and French sites suggest a higher content of shrubs in the diet of Holocene bison than in Pleistocene steppe bison. However, the diet of steppe bison was probably not totally free of woody vegetation, since some individuals, such as the bison bull mummy “Blue Babe” from Alaska (which lived in the Mid-Wisconsin warming interstadial around 36,425 + 2575/-1974 BP (QC-891)), had 7% of woody material among the plant fragments trapped in its teeth [106]. The diet of the steppe bison in our study was probably exclusively composed of grasses or sedges and forbs, due to the limitation of browse vegetation during the glacial period.

When compared with modern bison populations living in forested landscapes in Europe and North America, the Early Holocene bison exhibit the highest δ^13^C values. During the Early Holocene, the habitat use of bison was significantly different from that of all the extant forest bison populations, including those from boreal forest or those supplemented by hay during the winter. This supports the refugee status of the European bison as proposed by Kerley et al [17]. For both bison and aurochs, forested areas were probably marginal or sub-optimal habitats that allowed them to survive only at lower densities and led to a declining fitness as predicted by Fretwell’s model [107]. Indeed, a progressive disappearance of both species from large areas of Europe was observed during the Holocene [5], [108]. However, wide range of stable isotopes values recorded in aurochs and bison (combined Early Holocene and modern populations of the species) indicate plasticity of both species that probably allowed them to adapt to the environmental changes that took place over thousands of years and survive much longer than other species of mega herbivores.

## Conclusions

This study is one of the first examples of the application of conservation palaeobiology in Europe. As stated by Dietl and Flessa [109], the fossil record can be used to understand ecological and evolutionary responses of species to changes in their environment. Thus, comparison of the isotopic composition of early Holocene and modern material for European bison provides evidence essential for bison conservation management.

According to results of this study, the European bison of early Holocene north central Europe was living in a relatively open tundra-like environment. It consumed some browse and probably also lichen and it can therefore be defined as a mixed feeder. The study demonstrates also that the species exhibited some niche partitioning with aurochs and moose, but at the same time avoided competition by different use of resources and space. In the course of the Holocene, the spread of more densely forested landscape did not prevent this species, which was pre-adapted to consume browse, from surviving. However, it did increase the risk of its extinction, as predicted by the refugee species concept [17]. Understanding the pre-refugee ecology of bison and its ability to live in relatively open habitats may open new perspectives for reintroducing this endangered species in habitats other than forests, as suggested recently [17], [20]. It is questionable whether bison would be able to survive in forests in larger populations without human support. Forest habitats offer sufficient biomass of graze in summer, however, mainly browse in winter. In a forested area the bison needs access to highly productive open river valleys or meadows which offers a high biomass of dry fodder in winter. Without such an access, or without human support as currently offered [17], the species probably would not survive in forest habitats, or at least those that occur today in Europe. The introduction of bison to forest habitats sooner or later results in an expansion of their range to involve more open habitats [110] and concomitant damage to farm crops in surrounding areas [75]. In the light of this study, bison conservation management and reintroduction programme require improvement to take into account pre-refugee ecology of the species and its response to environmental changes in course of the Holocene. Inclusion of geohistorical data as proposed by Dietl and Flessa [109] in decision making process may provide a more scientifically robust basis for bison conservation policies than those dependent on short-term observations recognizing bison as a forest specialist.

## Supporting Information

S1 Table Isotopic analysis data of the collagen extracted from bones of moose, aurochs, and reindeer, selected from published studies. Radiocarbon dates were calibrated using OxCal v4.2.3 with IntCal13 atmospheric curve [83], [84]. The following chronozones are used Younger Dryas (12,900–11,600 cal BP), Preboreal (11,600–10,640 cal. BP). The information about datings was previously published by Aaris-Sørensen et al. [4], Noe-Nygaard [69] and Jessen et al. [70].
(DOC)

S2 Table List of isotopic measurements of *Bison priscus* and *Bos primigenius* from Pleistocene. The data have been published previously by Bocherens et al. [71], [72].
(DOC)

S3 Table Description of modern ecosystems inhabited by European bison *Bison bonasus* and American bison *Bison bison* and measured isotopic values for these species with corrections of δ^13^C values for the shift due to anthropogenic CO_2_ emissions. Corrections were made using the equation from Feng [81]. ∆^13^C atm- the difference in the atmospheric CO_2_ δ ^13^C value according to the date of the animal death, δ^13^C corrected- values correspond to the δ^13^C _coll_ values set to the same atmospheric CO_2_ δ^13^C of -6.429‰. Values of hair from *Bison bison* have been adjusted for collagen-hair carbon isotopic fractionation [40].
(DOC)

S1 Text Paleobiological tracking of herbivorous mammal paleoecology using carbon and nitrogen isotopes in bone collagen. (DOC)
