# Supplementary material for: European Bison as a Refugee Species? Evidence from Isotopic Data on Early Holocene Bison and Other Large Herbivores in Northern Europe
Source: PLoS One. 2015 Feb 11;10(2):e0115090. doi: 10.1371/journal.pone.0115090 (PMC4324907; doi:10.1371/journal.pone.0115090)
Supplement: S1 Table — Radiocarbon dates were calibrated using OxCal v4.2.3 with IntCal13 atmospheric curve [83], [84]. The following chronozones are used Younger Dryas (12,900–11,600 cal BP), Preboreal (11,600–10,640 cal. BP). The information about datings was previously published by Aaris-Sørensen et al. [4], Noe-Nygaard [69] and Jessen et al. [70]. (DOC) [file pone.0115090.s001.doc]

**Table S1. Isotopic analysis data of the collagen extracted** from bones of moose, aurochs, and reindeer, selected from published studies.

| **Lab-no** | **Species** | **Country** | **Site** | **Chronozone** | **Age (^14^C yr BP)** | **Calibrated age (cal yr BP)** | **C/N** | **δ^13^C (‰)** | **δ^15^N (‰)** | **Source** |
| --- | --- | --- | --- | --- | --- | --- | --- | --- | --- | --- |
| AAR15630 | *Alces alces* | Denmark | Lundby Mose | Younger Dryas | 10,127±33 | 11,804 ± 192 | - | -21.0 | - | Jessen et al. [70] |
| AAR15632 | *Alces alces* | Denmark | Lundby Mose | Younger Dryas | 10,119±30 | 11,747 ± 240 | - | -20.1 | - | Jessen et al. [70] |
| AAR5469 | *Alces alces* | Denmark | Lundby Mose | Preboreal | 9950±75 | 11,469 ± 245 | - | -21.9 | - | Jessen et al. [70] |
| AAR5470 | *Alces alces* | Denmark | Lundby Mose | Preboreal | 9930±70 | 11,457 ± 244 | - | -21.6 | - | Jessen et al. [70] |
| AAR5471 | *Alces alces* | Denmark | Lundby Mose | Preboreal | 9860±70 | 11,387 ± 216 | - | -22.2 | - | Jessen et al. [70] |
| AAR15635 | *Alces alces* | Denmark | Lundby Mose | Preboreal | 9585±50 | 10,939 ± 201 | - | -21.3 | - | Jessen et al. [70] |
| AAR15633 | *Alces alces* | Denmark | Lundby Mose | Preboreal | 9553±33 | 10,904 ± 183 | - | -22.6 | - | Jessen et al. [70] |
| AAR15631 | *Bos primigenius* | Denmark | Lundby Mose | Preboreal | 9585±30 | 10,931 ± 170 | - | -21.1 | - | Jessen et al. [70] |
| - | *Bos primigenius* | Denmark | Frobjerg Banker | Boreal | 9125± 45 | 10,309 ± 103 | 3.09 | -21.7 | 5.1 | Noe-Nygaard et al. [69] |
| - | *Bos primigenius* | Denmark | Alsonderup | Boreal | 9375±55 | 10,587 ± 155 | 3.07 | -19.0 | 6.1 | Noe-Nygaard et al. [69] |
| - | *Bos primigenius* | Denmark | Knabstrupgard | Preboreal | 9920±45 | 11,414 ± 187 | 3.11 | -21.8 | 4.3 | Noe-Nygaard et al. [69] |
| - | *Bos primigenius* | Denmark | Stokholtshuse | Preboreal | 9655±110 | 10,972 ± 275 | 2.89 | -20.7 | 4.5 | Noe-Nygaard et al. [69] |
| - | *Bos primigenius* | Denmark | Store Tastrup | Preboreal | 9970±90 | 11,512 ± 293 | 2.86 | -20.9 | 4.0 | Noe-Nygaard et al. [69] |
| K-4870 | *Rangifer tarandus* | Denmark | Sohjem, Bornholm | Younger Dryas | 10120±140 | 11,811 ± 565 | - | -18.2 | - | Aaris -Sorensen et al. [4] |
| K-4871 | *Rangifer tarandus* | Denmark | Strangegard, Bornholm | Younger Dryas | 10050±130 | 11,646 ± 415 | - | -18.4 | - | Aaris -Sorensen et al. [4] |
| K-7078 | *Rangifer tarandus* | Denmark | Grejsdalen, Vejle | Preboreal | 9950±70 | 11,468 ± 240 | - | -18.6 | - | Aaris -Sorensen et al. [4] |
| K-7077 | *Rangifer tarandus* | Denmark | Vedde, Soro | Preboreal | 9940±100 | 11,501 ± 306 | - | -19.3 | - | Aaris -Sorensen et al. [4] |
| K-4873 | *Rangifer tarandus* | Denmark | Lindegard, Bornholm | Preboreal | 9800±135 | 11,230 ± 480 | - | -18.0 | - | Aaris -Sorensen et al. [4] |
| K-7081 | *Rangifer tarandus* | Denmark | Karrebakstorp, Nastved | Preboreal | 9800±100 | 11,200 ± 411 | - | -21.7 | - | Aaris -Sorensen et al. [4] |
| K-7075 | *Rangifer tarandus* | Denmark | Groderup, Faborg | Preboreal | 9800±95 | 11,200 ± 407 | - | -21.3 | - | Aaris -Sorensen et al. [4] |
| K-7068 | *Rangifer tarandus* | Denmark | Vollerslev, Koge | Preboreal | 9720±95 | 11,016 ± 268 | - | -18.7 | - | Aaris -Sorensen et al. [4] |
| K-4872 | *Rangifer tarandus* | Denmark | Almindingen, Bornholm | Preboreal | 9720±135 | 11,134 ± 468 | - | -17.8 | - | Aaris -Sorensen et al. [4] |
| K-7070 | *Rangifer tarandus* | Denmark | Bornholm | Preboreal | 9660±85 | 10,988 ± 234 | - | -19.7 | - | Aaris -Sorensen et al. [4] |
| K-7076 | *Rangifer tarandus* | Denmark | Silkeborg | Preboreal | 9580±65 | 10,941 ± 221 | - | -19.2 | - | Aaris -Sorensen et al. [4] |
| K-7073 | *Rangifer tarandus* | Denmark | Linnet, Vejle | Preboreal | 9500±105 | 10,844 ± 323 | - | -19.9 | - | Aaris -Sorensen et al. [4] |
| K-7071 | *Rangifer tarandus* | Denmark | Lolland | Preboreal | 9410±75 | 10,744 ± 326 | - | -20.3 | - | Aaris -Sorensen et al. [4] |
| K-7072 | *Rangifer tarandus* | Denmark | Skavngard Mose, Viborg | Boreal | 9260±100 | 10,462 ± 226 | - | -18.8 | - | Aaris -Sorensen et al. [4] |
| K-7066 | *Rangifer tarandus* | Denmark | Jordrup, Kolding | Boreal | 9210±95 | 10,431 ± 219 | - | -19.5 | - | Aaris -Sorensen et al. [4] |
| K-7074 | *Rangifer tarandus* | Denmark | Risbanke, Ringsted | Boreal | 9180±80 | 10,390 ± 168 | - | -19.2 | - | Aaris -Sorensen et al. [4] |

Radiocarbon dates were calibrated using OxCal v4.2.3 with IntCal13 atmospheric curve [83], [84]. The following chronozones are used Younger Dryas (12,900 - 11,600 cal BP), Preboreal (11,600-10,640 cal. BP). The information about datings was previously published by Aaris-Sørensen et al. [4], Noe-Nygaard [69] and Jessen et al. [70].
