# Supplementary material for: European Bison as a Refugee Species? Evidence from Isotopic Data on Early Holocene Bison and Other Large Herbivores in Northern Europe
Source: PLoS One. 2015 Feb 11;10(2):e0115090. doi: 10.1371/journal.pone.0115090 (PMC4324907; doi:10.1371/journal.pone.0115090)
Supplement: S2 Table — The data have been published previously by Bocherens et al. [71], [72]. (DOC) [file pone.0115090.s002.doc]

| **Lab-no** | **Species** | **Country** | **Site** | **Habitat type** | **^14^C dating (BP)** | **C/N** | **δ ^13^C (‰)** | **δ ^15^N (‰)** | **Source** |
| --- | --- | --- | --- | --- | --- | --- | --- | --- | --- |
| Goyet-A3-13 | *Bison priscus* | Belgium | Goyet cave | fork of the Samson River and the Meuse River | 28-40,000 | - | -20.0 | 4.1 | Bocherens et al. [71] |
| Goyet-A3-14 | *Bison priscus* | Belgium | Goyet cave | fork of the Samson River and the Meuse River | 28-40,000 | - | -20.4 | 4.8 | Bocherens et al. [71] |
| Goyet-B4-5 | *Bison priscus* | Belgium | Goyet cave | fork of the Samson River and the Meuse River | 28-40,000 | - | -20.0 | 5.7 | Bocherens et al. [71] |
| Goyet-B4-7 | *Bison priscus* | Belgium | Goyet cave | fork of the Samson River and the Meuse River | 28-40,000 | - | -20.4 | 3.9 | Bocherens et al. [71] |
| SC29000 | *Bison priscus* | Belgium | Scladina cave | valley of the Meuse River | 40,000 | - | -20.8 | 4.4 | Bocherens et al. [71] |
| SC29100 | *Bison priscus* | Belgium | Scladina cave | valley of the Meuse River | 40,000 | - | -19.8 | 5.6 | Bocherens et al. [71] |
| Goyet-B4-6 | *Bos primigenius* | Belgium | Goyet cave | fork of the Samson River and the Meuse River | 28-40,000 | - | -20.0 | 3.8 | Bocherens et al. [71] |
| SC28800 | *Bos primigenius* | Belgium | Scladina cave | valley of the Meuse River | 40,000 | - | -20.7 | 4.9 | Bocherens et al. [71] |
| SC28900 | *Bos primigenius* | Belgium | Scladina cave | valley of the Meuse River | 40,000 | - | -20.3 | 4.5 | Bocherens et al. [71] |
| LBR100 | *Bison priscus* | France | La Berbie | L'Isle River valley | 35,000 | 3.2 | -20.3 | 6.1 | Bocherens et al. [72] |
| LBR200 | *Bison priscus* | France | La Berbie | L'Isle River valley | 35,000 | 3.2 | -20.1 | 5.6 | Bocherens et al. [72] |
| LBR300 | *Bison priscus* | France | La Berbie | L'Isle River valley | 35,000 | 3.1 | -20.8 | 4.7 | Bocherens et al. [72] |
| LBR400 | *Bison priscus* | France | La Berbie | L'Isle River valley | 35,000 | 3.1 | -20.3 | 5.6 | Bocherens et al. [72] |
| LBR500 | *Bison priscus* | France | La Berbie | L'Isle River valley | 35,000 | 3.2 | -20.2 | 6.1 | Bocherens et al. [72] |
| LBR600 | *Bison priscus* | France | La Berbie | L'Isle River valley | 35,000 | 3.0 | -20.2 | 6.0 | Bocherens et al. [72] |
| LBR700 | *Bison priscus* | France | La Berbie | L'Isle River valley | 35,000 | 3.1 | -20.9 | 4.6 | Bocherens et al. [72] |
| LBR3100 | *Bison priscus* | France | La Berbie | L'Isle River valley | 35,000 | 3.0 | -20.5 | 5.3 | Bocherens et al. [72] |
| CAM100 | *Bos primigenius* | France | Camiac | Garonne River valley | 35,000 | 3.2 | -20.0 | 6.1 | Bocherens et al. [72] |
| CAM200 | *Bos primigenius* | France | Camiac | Garonne River valley | 35,000 | 3.2 | -20.7 | 6.2 | Bocherens et al. [72] |
| CAM300 | *Bos primigenius* | France | Camiac | Garonne River valley | 35,000 | 3.3 | -20.4 | 7.4 | Bocherens et al. [72] |
| CAM400 | *Bos primigenius* | France | Camiac | Garonne River valley | 35,000 | 3.2 | -20.1 | 8.6 | Bocherens et al. [72] |
| CAM500 | *Bos primigenius* | France | Camiac | Garonne River valley | 35,000 | 3.3 | -20.0 | 6.0 | Bocherens et al. [72] |
| CAM600 | *Bos primigenius* | France | Camiac | Garonne River valley | 35,000 | 3.3 | -20.4 | 5.3 | Bocherens et al. [72] |

**Table S2. List of isotopic measurements of *Bison* *priscus* and *Bos primigenius* from Pleistocene.**

The data have been published previously by Bocherens et al. [71], [72].
