# Supplementary material for: European Bison as a Refugee Species? Evidence from Isotopic Data on Early Holocene Bison and Other Large Herbivores in Northern Europe
Source: PLoS One. 2015 Feb 11;10(2):e0115090. doi: 10.1371/journal.pone.0115090 (PMC4324907; doi:10.1371/journal.pone.0115090)
Supplement: S3 Table — Corrections were made using the equation from Feng [81]. ∆13C atm- the difference in the atmospheric CO2 δ 13C value according to the date of the animal death, δ13C corrected- values correspond to the δ13C coll values set to the same atmospheric CO2 δ13C of -6.429‰. Values of hair from Bison bison have been adjusted for collagen-hair carbon isotopic fractionation [40]. (DOC) [file pone.0115090.s003.doc]

**Table S3. Description of modern ecosystems inhabited by European bison *Bison bonasus* and American bison *Bison bison* and measured isotopic values for these species with corrections of δ^13^C values for the shift due to anthropogenic CO_2_** emissions.

| **Lab-no** | **Species** | **Country** | **Region** | **Habitat** | **Year of death** | **δ^13^C _coll_ (‰)** | **∆^13^C atm** | **δ^13^C corrected** | **Reference** |
| --- | --- | --- | --- | --- | --- | --- | --- | --- | --- |
| 59 BC | *Bison bonasus* | Poland | Bieszczady Mountains | Deciduous and mixed forest, mountain, utilized elevation 450-950 m.a.s.l., low use of winter supplementation | 2004 | -25.3 | 1.85 | -23.4 | This paper |
| 60 BC | *Bison bonasus* | Poland | Bieszczady Mountains | Deciduous and mixed forest, mountain, utilized elevation 450-950 m.a.s.l., low use of winter supplementation | 2004 | -25.1 | 1.85 | -23.3 | This paper |
| 61 BC | *Bison bonasus* | Poland | Bieszczady Mountains | Deciduous and mixed forest, mountain, utilized elevation 450-950 m.a.s.l., low use of winter supplementation | 2009 | -25.9 | 2.06 | -23.9 | This paper |
| 62 BC | *Bison bonasus* | Poland | Bieszczady Mountains | Deciduous and mixed forest, mountain, utilized elevation 450-950 m.a.s.l., low use of winter supplementation | 2011 | -25.8 | 2.15 | -23.6 | This paper |
| 88 BC | *Bison bonasus* | Poland | Bieszczady Mountains | Deciduous and mixed forest, mountain, utilized elevation 450-950 m.a.s.l., low use of winter supplementation | 2011 | -25.7 | 2.15 | -23.6 | This paper |
| BLW100 | *Bison bonasus* | Poland | Białowieża Forest | Deciduous and mixed forest, plain, high winter supplementation | 1987 | -23.2 | 1.28 | -21.9 | Drucker et al. [40] |
| BLW300 | *Bison bonasus* | Poland | Białowieża Forest | Deciduous and mixed forest, plain, high winter supplementation | 1998 | -23.0 | 1.62 | -21.4 | Drucker et al. [40] |
| BLW400 | *Bison bonasus* | Poland | Białowieża Forest | Deciduous and mixed forest, plain, high winter supplementation | 1998 | -23.7 | 1.62 | -22.1 | Drucker et al. [40] |
| BLW500 | *Bison bonasus* | Poland | Białowieża Forest | Deciduous and mixed forest, plain, high winter supplementation | 1999 | -24.4 | 1.66 | -22.7 | Drucker et al. [40] |
| BLW600 | *Bison bonasus* | Poland | Białowieża Forest | Deciduous and mixed forest, plain, high winter supplementation | 1999 | -25.6 | 1.66 | -23.9 | Drucker et al. [40] |
| ACT400 | *Bison bonasus* | Poland | Białowieża Forest | Deciduous and mixed forest, plain, high winter supplementation | 1994 | -23.6 | 1.49 | -22.1 | Drucker et al. [40] |
| 17270 | *Bison bison* | Canada | Prince Albert National Park | Boreal forest, plain, no winter supplementation | 2002 | -23.1 | 1.77 | -21.3 | Drucker et al. [40] |
| 17271 | *Bison bison* | Canada | Prince Albert National Park | Boreal forest, plain, no winter supplementation | 2002 | -21.5 | 1.77 | -19.7 | Drucker et al. [40] |
| 17272 | *Bison bison* | Canada | Prince Albert National Park | Boreal forest, plain, no winter supplementation | 2002 | -22.7 | 1.77 | -20.9 | Drucker et al. [40] |
| 19969 | *Bison bison* | Canada | Prince Albert National Park | Boreal forest, plain, no winter supplementation | 2002 | -23.4 | 1.77 | -21.6 | Drucker et al. [40] |
| 19970 | *Bison bison* | Canada | Prince Albert National Park | Boreal forest, plain, no winter supplementation | 2002 | -24.3 | 1.77 | -22.5 | Drucker et al. [40] |
| 19972 | *Bison bison* | Canada | Prince Albert National Park | Boreal forest, plain, no winter supplementation | 2002 | -24.6 | 1.77 | -22.8 | Drucker et al. [40] |
| 19973 | *Bison bison* | Canada | Prince Albert National Park | Boreal forest, plain, no winter supplementation | 2002 | -23.7 | 1.77 | -21.9 | Drucker et al. [40] |
| 19974 | *Bison bison* | Canada | Prince Albert National Park | Boreal forest, plain, no winter supplementation | 2002 | -23.9 | 1.77 | -22.1 | Drucker et al. [40] |
| 19975 | *Bison bison* | Canada | Prince Albert National Park | Boreal forest, plain, no winter supplementation | 2002 | -23.9 | 1.77 | -22.1 | Drucker et al. [40] |

Corrections were made using the equation from Feng [81]. ∆^13^C atm- the difference in the atmospheric CO_2_ δ^13^C value according to the date of the animal death, δ^13^C corrected- values correspond to the δ^13^C _coll_ values set to the same atmospheric CO_2_ δ^13^C of -6.429‰. Values of hair from *Bison bison* have been adjusted for collagen-hair carbon isotopic fractionation [40].
