# Supplementary material for: European Bison as a Refugee Species? Evidence from Isotopic Data on Early Holocene Bison and Other Large Herbivores in Northern Europe
Source: PLoS One. 2015 Feb 11;10(2):e0115090. doi: 10.1371/journal.pone.0115090 (PMC4324907; doi:10.1371/journal.pone.0115090)
Supplement: S1 Text — (DOC) [file pone.0115090.s004.doc]

## Text S1. Paleobiological tracking of herbivorous mammal paleoecology using carbon and nitrogen isotopes in bone collagen.

Bones are composed of both organic and mineral fractions which are synthesized during the lifetime of a vertebrate. The organic fraction of bone is mainly formed of a protein, collagen, which contains carbon (around 40%) and nitrogen (around 15%). The isotopic signatures of carbon and nitrogen are measured in the collagen organic fraction. In fossil bone material, carbon and nitrogen isotopic values are unchanged despite the post-mortem processes when the carbon and nitrogen amounts of residual collagen remain in the range of those observed in collagen extracted from fresh bone using the same technique [79], [80].

**Carbon**

All the carbon of an organism comes from its dietary intake, in the form of proteins, carbohydrates and lipids. Some of these nutrients are incorporated directly by the organism and sequestered in different tissues, while other molecules are synthesized by the organism from dietary nutrients. Due to these different biochemical characteristics and isotopic fractionations, the average carbon isotopic abundance of a vertebrate is close to that of its average diet, but those recorded in a given tissue or molecule exhibit specific differences. The δ^13^C values of mammal bone collagen are typically 5‰ more positive than those of the average diet. Therefore, the δ^13^C values of collagen are typically used to track the type of plant food consumed by herbivores.

The δ^13^C values discriminate between different types of plants, principally between marine and terrestrial plants. Among terrestrial plants, the carbon isotope signature varies between plants using the two main photosynthetic pathways, called C_3_ and C_4_ ("C_3_–plants" and "C_4_–plants"). C_4_ plants are absent or very limited in environments with a mild or cold growing season, as in Europe. In environments where all plants use the C_3_ photosynthetic pathway, like in temperate and cold regions of Europe, an isotopic distinction can be seen between plants growing under a closed canopy and those at the top of the canopy or growing in an open environment. The possible causes of the so-called “canopy effect” are the concentration of recycled CO_2_ due to poor ventilation, the light attenuation and the relative high water availability in closed canopy forest (e.g. Gebauer and Schultze [38]; van der Merwe and Medina [35]; Broadmeadow et al. [36]). Since herbivore bones record the δ^13^C values of their plant food, it is possible to identify which kind of plant was consumed by an herbivore, and therefore the type of environment in which it lived.

**Nitrogen**

Nitrogen is incorporated through dietary intake in the organic molecules of an organism's tissues. It is usually measured in the collagen preserved in fossil bones. Contrarily to carbon, the isotopic signature of nitrogen is significantly enriched in vertebrate tissues relatively to its average diet, typically by 3 to 5‰. Therefore, the nitrogen isotopic signature of a given individual herbivore depends on the isotopic signature at the base of the foodweb to which it belongs, i.e. in the consumed plants. Not all herbivores present the same δ^15^N values in a given ecosystem since different plants may use nitrogen under different forms, leading to varying isotopic fractionation and δ^15^N values. For instance, grass and graminoids typically exhibit more positive δ^15^N values than shrubs and trees, as the latter obtain their nitrogen through symbiotic fungal mycorrhizae (e.g. Schulze et al. [49]; Michelsen et al. [55]). Global climatic factors such as aridity and temperature lead to increased δ^15^N values of plants [57], while δ^15^N of plants tend to decrease with increasing altitude.
